# Pro-Cellular Exhaustion Markers are Associated with Splenic Microarchitecture Disorganization and Parasite Load in Dogs with Visceral Leishmaniasis

**DOI:** 10.1038/s41598-019-49344-1

**Published:** 2019-09-10

**Authors:** Tainã Luís de Souza, Aurea Virginia Andrade da Silva, Luiza de Oliveira Ramos Pereira, Fabiano Borges Figueiredo, Artur Augusto Velho Mendes Junior, Rodrigo Caldas Menezes, Daniella Areas Mendes-da-Cruz, Mariana Côrtes Boité, Elisa Cupolillo, Renato Porrozzi, Fernanda Nazaré Morgado

**Affiliations:** 1 Laboratório de Pesquisa em Leishmaniose, Instituto Oswaldo Cruz, Fundação Oswaldo Cruz, Rio de Janeiro, Brazil; 2 Laboratório de Biologia Celular, Instituto Carlos Chagas, Fundação Oswaldo Cruz, Paraná, Brazil; 3 Laboratório de Pesquisa Clínica em Dermatozoonoses em Animais Domésticos, Instituto Nacional de Infectologia Evandro Chagas, Fundação Oswaldo Cruz, Rio de Janeiro, Brazil; 4 Laboratório de Pesquisas Sobre o Timo, Instituto Oswaldo Cruz, Fundação Oswaldo Cruz, Rio de Janeiro, Brazil

**Keywords:** Parasite host response, Cellular immunity

## Abstract

In canine visceral leishmaniasis (CVL), splenic white pulp (SWP) disorganization has been associated with disease progression, reduced cytokine and chemokine expression and failure to control the parasite load. This profile is compatible with the cellular exhaustion previously shown in human visceral leishmaniasis. The present study aimed to evaluate the *in situ* expression of cellular exhaustion markers and their relation to clinical signs, SWP disorganization and parasite load. Forty dogs naturally infected by *Leishmania infantum* were grouped according to levels of SWP organization and parasite load. SWP disorganization was associated with reductions in the periarteriolar lymphatic sheath and lymphoid follicles/mm^2^ and worsening of the disease. Apoptotic cells expressing CTLA-4^+^ increased in dogs with disorganized SWP and a high parasite load. In the same group, PD-L1 and LAG-3 gene expression were reduced. A higher number of CD21^+^TIM-3^+^ B cells was detected in disorganized spleens than in organized spleens. Apoptosis is involved in periarteriolar lymphatic sheath reduction and lymphoid follicle atrophy and is associated with CTLA-4^+^ cell reductions in the splenic tissue of dogs with visceral leishmaniasis (VL). Failure to control the parasite load was observed, suggesting that cell exhaustion followed by T and B cell apoptosis plays a role in the immunosuppression observed in CVL.

## Introduction

Zoonotic visceral leishmaniasis (ZVL) is a tropical and subtropical disease caused by *Leishmania infantum*, an intracellular protozoan that is transmitted to its vertebrate hosts by the blood feeding of female sand flies of the genera Phlebotomus and *Lutzomyia*. Dogs have been implicated as the main urban reservoir of *L*. *infantum* and source of infection for the vector *Lutzomyia longipalpis*, the known vector in South America. Infected dogs present high parasite loads in the skin and blood^1^ and are major targets for control measures. Furthermore, dogs are considered good models for understanding the immunopathogenesis of ZVL^2^.

*L*. *infantum* infection affects several organs in dogs, and the spleen is an important target. Notably, disorganization of the splenic white pulp (SWP) has been reported in naturally infected dogs^3–7^. However, little is known about the mechanisms triggering splenic disorganization or the influence of parasites or the immune response on this process. The maintenance of the splenic microarchitecture and areas of segregation in the spleen is important for the activation of effector lymphocytes and for the development of specific immune responses^8,9^. It has been generally demonstrated that the progression of an *L*. *infantum* infection to active disease is characterized by a marked humoural response, depression of the cellular response against the parasite, and the emergence of clinical signs^2^. Recent studies have correlated the progression of disease with the disorganization of the splenic microarchitecture^3,10^, leading to increased parasite load and reduced expression of cytokines, chemokines and chemokine receptors^11^, which is compatible with the cellular exhaustion profile.

T cell exhaustion is defined by decreased effector function, sustained expression of inhibitory receptors and a transcriptional state distinct from that of functional effector or memory T cells^12^. Cellular exhaustion characterized by programmed death 1 (PD-1) expression was first described in viral infection^13^. PD-1 expression is induced by repeated antigenic stimulation in T and B lymphocytes, and this non-responsive state is termed exhaustion^14^. The ligand of PD-1, PD-L1, is constitutively expressed by B lymphocytes, T lymphocytes, macrophages and dendritic cells in the spleen^14^. Activation of PD-1 induces apoptosis and inhibits cell proliferation as well as cytokine production^15^. It was further demonstrated that exhaustion could be reversed *in vivo* and *in vitro* by the administration of antibodies specific to the ligand (PD-L1), leading to recovery of the proliferative capacity of CD8^+^ cells, secretion of cytokines, elimination of infected cells and reduction in the viral load^13^. The expression of PD-1 and cytotoxic T lymphocyte antigen 4 (CTLA-4) by peripheral and splenic CD8^+^ cells has been demonstrated in human visceral leishmaniasis (VL)^16^ and in mice experimentally infected with *Leishmania donovani*, another *Leishmania* species that causes VL^15–17^. Administration of anti-CTLA-4 blocking antibodies *in vivo* led to increased frequencies of IFN-γ- and IL-4-producing cells in the liver and spleen of the experimentally infected mice and accelerated the development of the hepatic granulomatous response associated with a reduction in the parasite load^17^. The emergence of exhausted CD8^+^ cells was accompanied by a reduction in inflammatory cytokine levels^15^. It has also been shown that receptor blockade does not influence IFN-γ production; however, in a murine experimental model, PD-L1 (B7-H1) blockade led to control of the parasite load even with unchanged induction of cytokine production^15^. In an evaluation of splenic cells from patients with VL, blocking the PD-1/PD-L1 or CTLA-4 pathways did not alter IFN-γ production or parasite survival *in vitro*^16^. We hypothesized that failures in mounting an effective immune response and in controlling the parasite load are associated with the disorganization of the SWP and cellular exhaustion in dogs presenting with VL. Thus, we evaluated the expression of the exhaustion markers PD-1, PD-L1, CTLA-4, TIM-3 and LAG-3 in the spleen of dogs naturally infected with *L*. *infantum* and correlated the results with the clinical signs, organization of the SWP and parasite load observed in the animals.

## Results

### Associations among clinical score, parasite load and SWP disorganization in dogs naturally infected with *L*. *infantum*

In the present study, 40 dogs naturally infected with *L*. *infantum* were included. The six clinical signs most frequently observed in the dogs with ZVL were evaluated: onychogryphosis, keratoconjunctivitis, dermatitis, body condition score, lymphadenomegaly and alopecia. Considering the intensity of the clinical signs, the animals were grouped into low (n = 13), medium (n = 13) and high clinical score (n = 14) groups. According to the degree of SWP organization and the parasite load, the dogs were grouped as follows: 1- organized SWP and low parasite load (OL; n = 10); 2- disorganized SWP and low parasite load (DL; n = 22); and 3- disorganized SWP and high parasite load (DH; n = 8).

The disorganization of the SWP was characterized by a reduction in the number and loss of compartmentalization of the lymphoid follicles, which were atrophied in the dogs with severe ZVL (Supplementary Fig. S1). Moreover, there was a reduction in cell number in the periarteriolar lymphatic sheath, an area composed of T lymphocytes. This histopathological sign was observed in most animals (30/40, 75%). Furthermore, this alteration was associated with disease progression since the dogs with disorganized SWP showed significantly higher clinical scores than those with organized SWP (Mann-Whitney p = 0.0235) (Supplementary Fig. S1E).

### Qualitative and quantitative analysis of the CD4^+^, CD8^+^ and CD21^+^ cells in the spleen of dogs naturally infected with *L*. *infantum*

**Figure 1:**
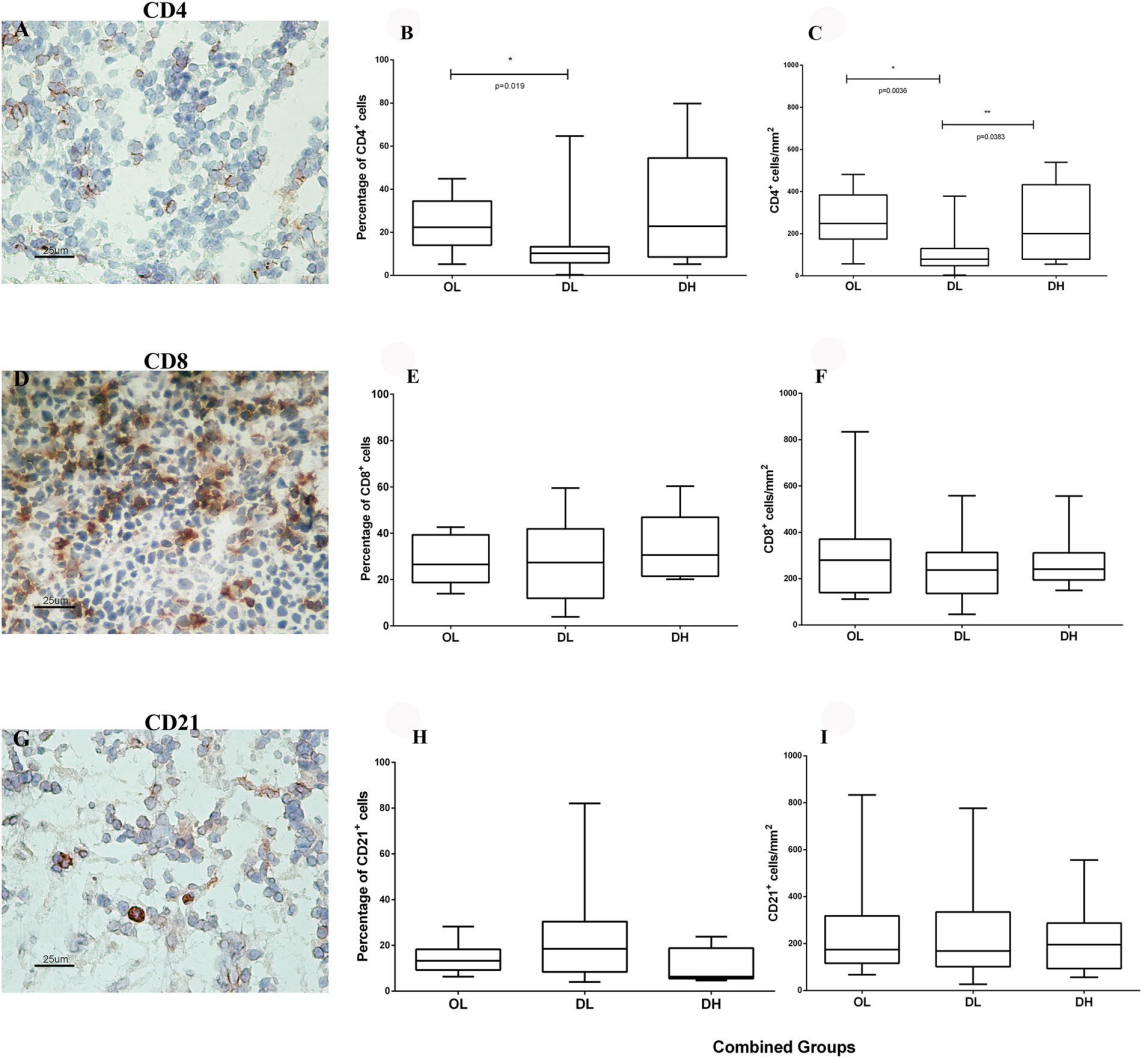
Identification of (**A**–**C**) CD4^+^, (**D**–**F**) CD8^+^, and (**G**–**I**) CD21^+^ cells by immunohistochemistry and quantitative analysis of the spleen of dogs naturally infected with *L*. *infantum* grouped according to the disorganization of the splenic white pulp and the parasite load: (**B**) percentage of CD4^+^ cells, (**C**) CD4^+^ cells/mm^2^, (**E**) percentage of CD8^+^ cells, (**F**) CD8^+^ cells/mm^2^, (**H**) percentage of CD21^+^ cells, and (**I**) CD21^+^ cells/mm^2^. Mann-Whitney test: (**B**) *p = 0.019; (**C**) *p = 0.0036, **p = 0.0383. Kruskal-Wallis test: (**B**) p = 0,031; (**C**) p = 0.021.

CD4^+^, CD8^+^ and CD21^+^ cells were detected in the red and white pulps of the spleen by immunohistochemistry (Fig. 1A,D,G). Cellular quantification was performed in the red pulp, as it is a cell transition area and the site where parasitized macrophages are concentrated. It should be noted that follicle size may vary even in the same analysed field, and several animals did not present follicles in the splenic sections, justifying the use of the red pulp for these analyses.

CD4^+^ and CD8^+^ cells were concentrated in the periarteriolar lymphoid sheath and homogeneously distributed in the red pulp, whereas CD21^+^ cells were more concentrated in the lymphoid follicles. Lower numbers of CD4^+^ cells were observed in the DL animals than in the DH animals. In association with periarteriolar lymphoid sheath atrophy, these data suggested the retention of these cells in the red pulp (Fig. 1B,C). No differences were observed in the numbers of CD8^+^ and CD21^+^ cells among the OL, DL and DH groups (Fig. 1E,F,H,I). In addition, there were no significant differences in the cellular profile when the OL, DL and DH groups were compared according to clinical score (Supplementary Fig. S2).

### Qualitative and quantitative analysis of cytokine expression (IFN-γ and IL-10) and cell proliferation (Ki-67) in the spleen of dogs naturally infected with *L*. *infantum*

**Figure 2:**
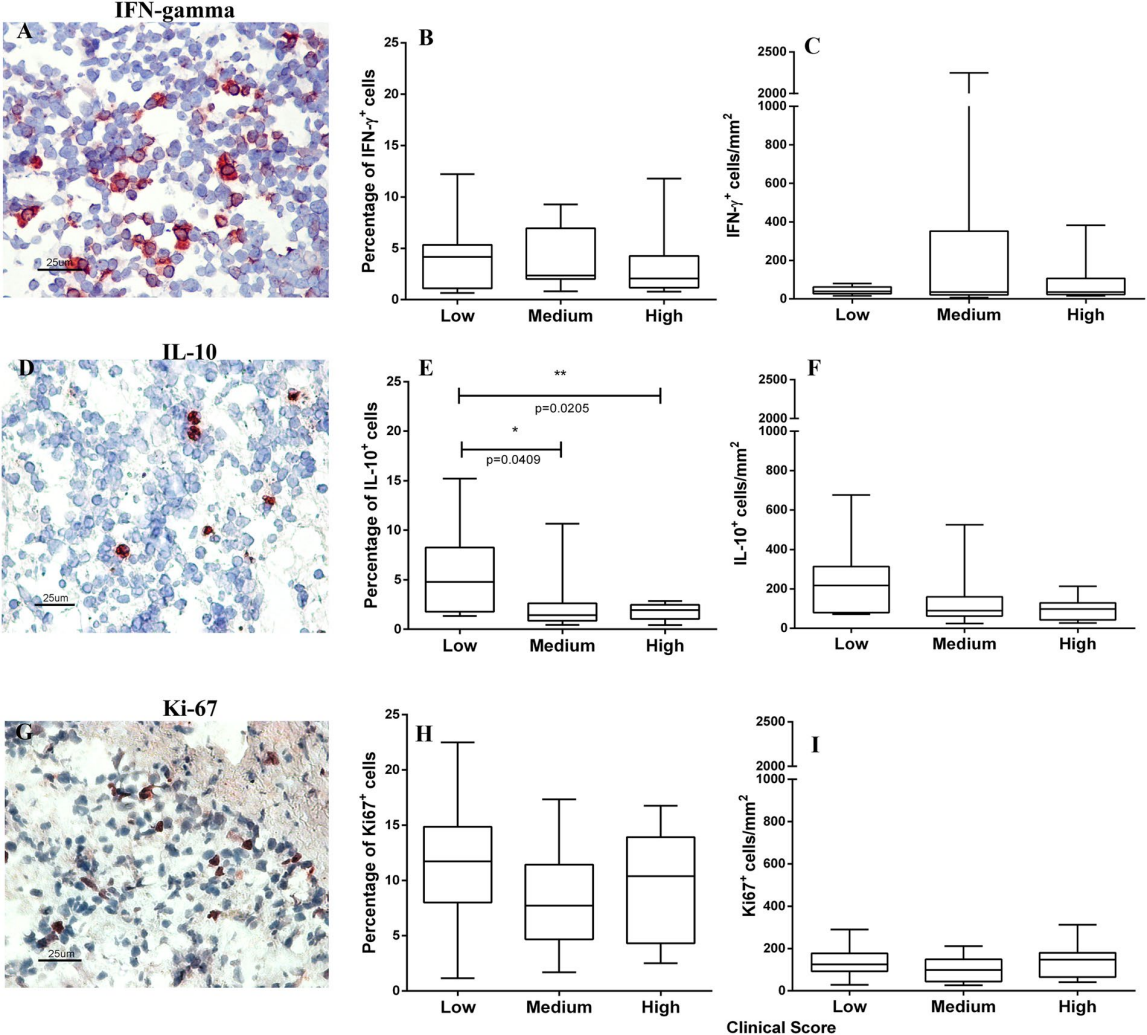
Identification of (**A**–**C**) IFN-γ^+^, (**D**–**F**) IL-10^+^, and (**G**–**I**) Ki-67^+^ cells by immunohistochemistry and quantitative analysis of the spleen of dogs naturally infected with *L*. *infantum* grouped according to the clinical score: (**B**) percentage of IFN-γ^+^ cells, (**C**) IFN-γ cells/mm^2^, (**E**) percentage of IL-10^+^ cells, (**F**) IL-10^+^ cells/mm^2^, (**H**) percentage of Ki-67^+^ cells and (**I**) Ki-67^+^ cells/mm^2^. Mann-Whitney test: (**E**) *p = 0.0409, **p = 0.0205. Kruskal-Wallis test: (**E**) p = 0.026.

IFN-γ^+^, IL-10^+^ and Ki-67^+^ cells were detected in the spleen of naturally infected dogs (Fig. 2A,D,G). These cells were homogeneously distributed in the splenic red pulp and were poorly identified in the white pulp. When the groups stratified according to SWP organization and parasite load were compared, no significant differences in the numbers of IFN-γ^+^, IL-10^+^ and Ki-67^+^ cells were observed (Supplementary Fig. S3).

Further comparison of these three groups revealed no difference in the number of IFN- γ^+^ cells (Fig. 2B,C) or in the number of Ki-67^+^ cells (Fig. 2H–I). However, fewer IL-10^+^ cells were observed in the animals with the highest clinical score (Fig. 2E,F).

### Qualitative and quantitative analysis of TIM-3, CTLA-4, PD-1, PD-L1, PD-L2, and LAG-3 expression in the spleen of dogs naturally infected with *L*. *infantum*

**Figure 3:**
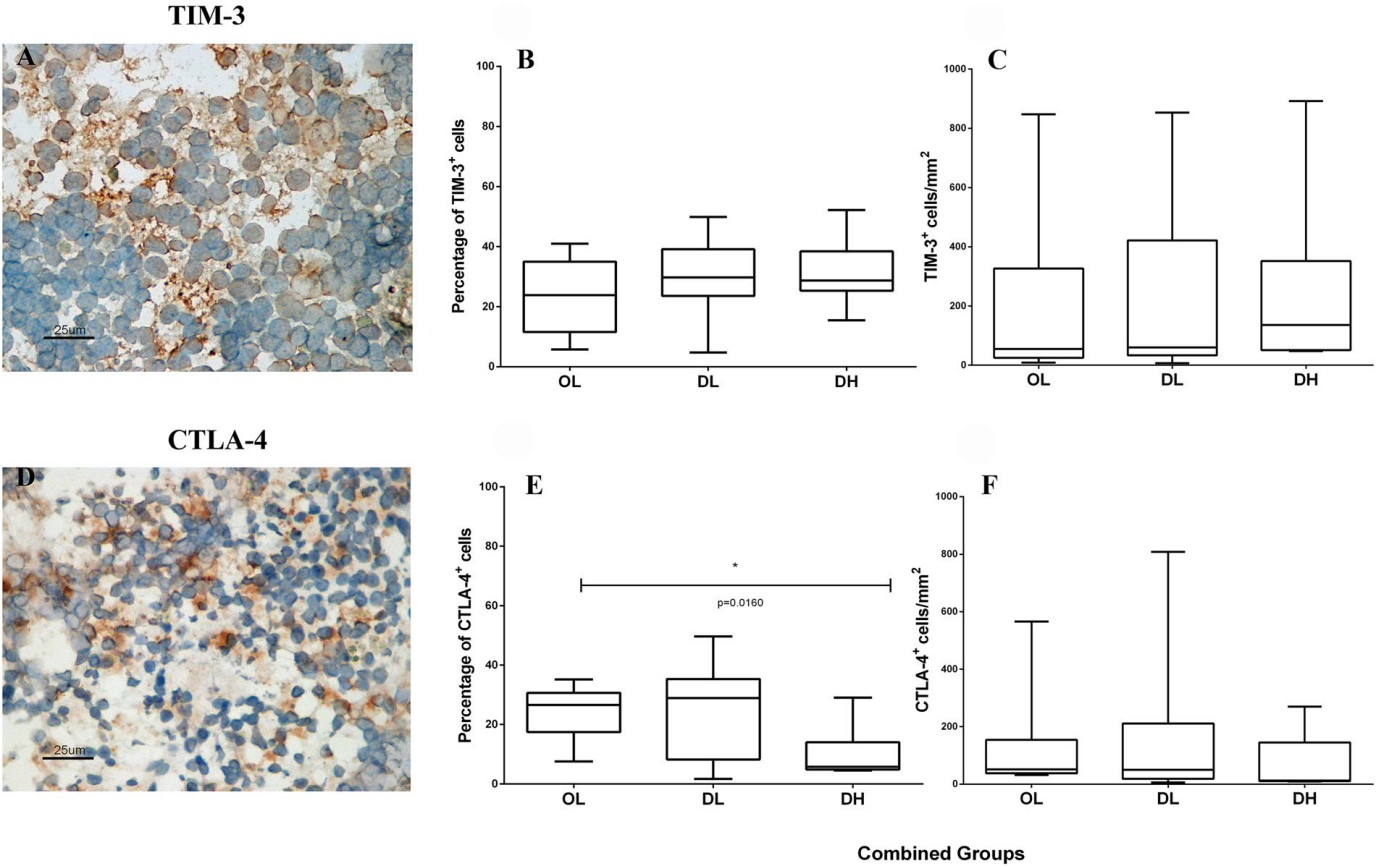
Identification of (**A**–**C**) TIM-3^+^ and (**D**–**F**) CTLA-4^+^ cells by immunohistochemistry and quantitative analysis of exhausted cells in the spleen of dogs naturally infected with *L*. *infantum* grouped according to the disorganization of the splenic white pulp and the parasite load: (**B**) percentage of TIM-3^+^ cells, (**C**) TIM-3 cells/mm^2^, (**E**) percentage of CTLA-4^+^ cells, and (**F**) CTLA-4^+^ cells/mm^2^. Mann-Whitney test: (**E**) *p = 0.00160. Kruskal-Wallis test: (**E**) p = 0.021.

TIM-3^+^ and CTLA-4^+^ cells were detected in the spleen of each naturally infected dog (Fig. 3A,D). These cells were more abundant in the splenic red pulp and were homogeneously distributed in this region. Animals in the OL and DL groups showed more CTLA-4^+^ cells than did those in the DH group (Fig. 3). We also observed that TIM-3 expression was high in all groups, and, although not significant, TIM-3^+^ cell numbers were higher in animals with disorganized SWP and those with high parasite loads than in the other animals (Fig. 3B,C). Furthermore, animals with more intense clinical scores presented higher numbers of TIM-3^+^ cells than did those with low clinical scores (Supplementary Fig. S4A,B). There were no differences between the clinical groups when comparing the numbers of CTLA-4^+^ cells (Supplementary Fig. S4C,D).

**Figure 4:**
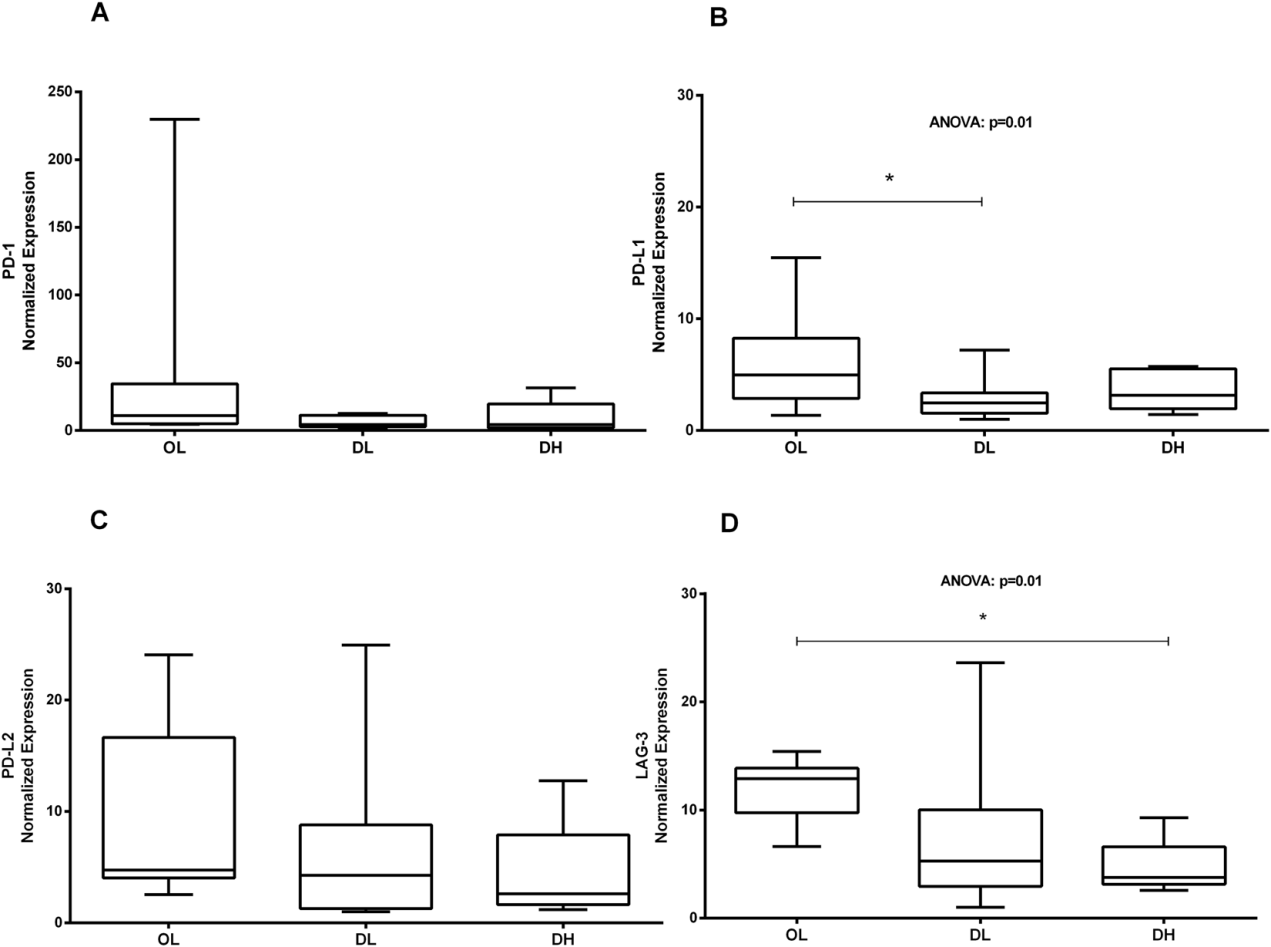
Gene expression of exhaustion markers in the spleen of dogs naturally infected with *L*. *infantum*. *Ex vivo* analysis by qPCR of the mRNA levels in the spleen of dogs classified according to the parasite load and splenic disorganization. Gene expression values were normalized to those of the constitutive genes HPRT and GAPDH. ANOVA: (**B**) p = 0.01; (**D**) p = 0.01. Tukey’s test (*): (**B**) p < 0.05; (**D**) p < 0.05.

**Figure 5:**
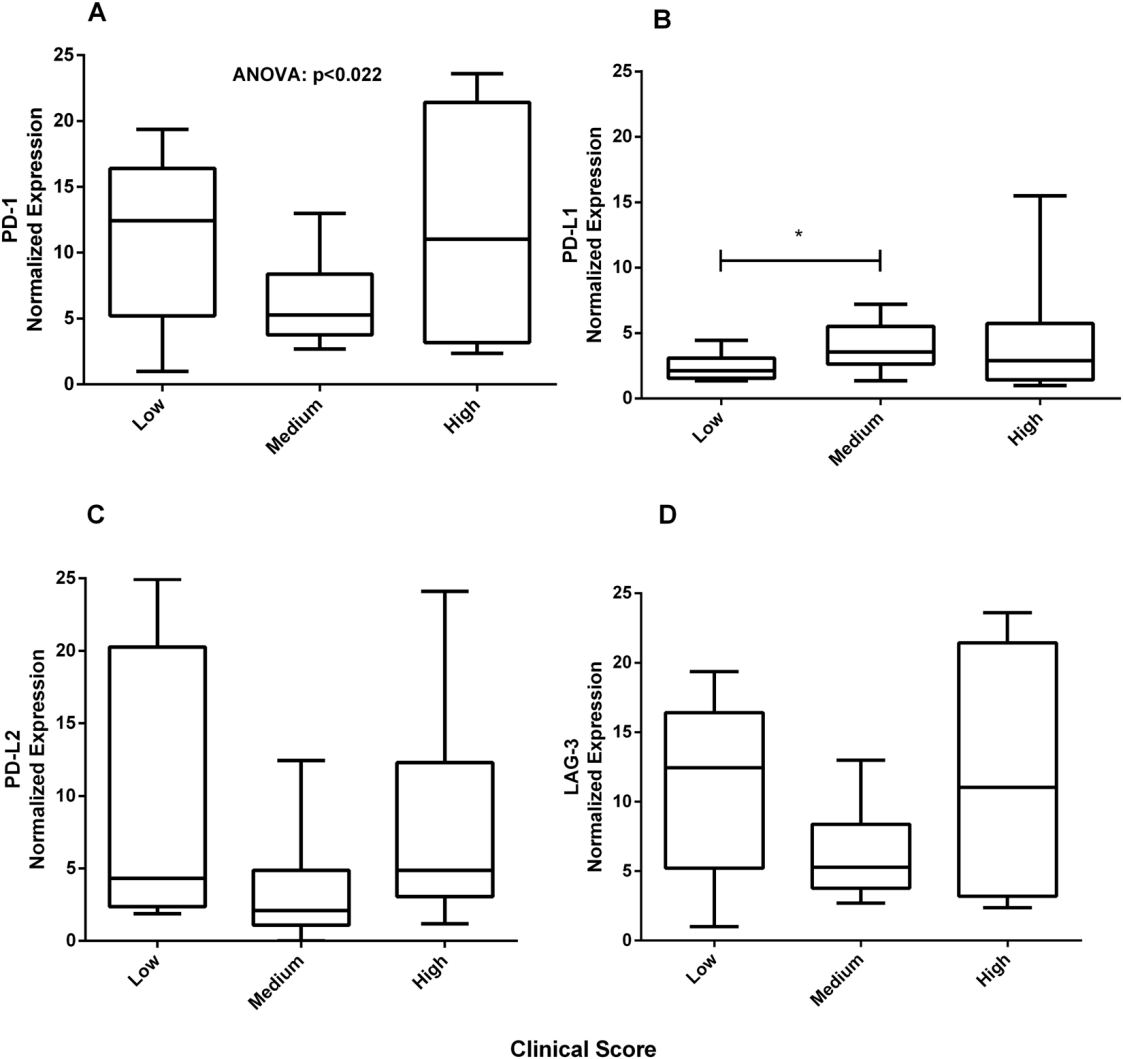
Gene expression of exhaustion markers in the spleen of dogs naturally infected with *L*. *infantum*. *Ex vivo* analysis by qPCR of the mRNA levels in the spleen of dogs classified according to the clinical score. Gene expression values were normalized to those of the constitutive genes HPRT and GAPDH. ANOVA: (**A**) p < 0.022. (*) Mann-Whitney test: (**B**) p < 0.036.

PD-1^+^ and LAG-3^+^ cells could not be identified by immunohistochemistry in this study, perhaps due to the limited ability of this technique to analyse molecules with low expression. Therefore, the gene expression of these exhaustion markers plus PD-L1 and PD-L2 was assayed by qRT-PCR (Fig. 4). The expression of the four markers was reduced in the groups that presented greater disorganization and higher parasite load in the spleen; significant differences were found for PD-L1 and LAG-3. In addition, PD-L1 expression was lower in the groups that presented low clinical scores than in the groups with intermediate or high clinical scores (Fig. 5).

### Quantitative analysis of CD4^+^ and CD8^+^ subpopulations expressing the exhaustion receptors TIM-3 and CTLA-4

Splenocytes were isolated from animals in the OL (n = 5; Media 8.2 × 10^6^, min-max 2.8–14.0 × 10^6^ cells/ml), DL (n = 5; Media 11.1 × 10^6^, min-max 2.5–21.5 × 10^6^ cells/ml) and DH (n = 5; Media 4.95 × 10^6^, min-max 3.8–6.1 × 10^6^ cells/ml) groups.

We detected CD4^+^ and CD8^+^ cells expressing both exhaustion receptors (TIM-3 and CTLA-4) (Supplementary Fig. S5). Although we did not observe significant differences among the groups, the DL group presented the highest CD4^+^CTLA-4^+^ counts, similar to that observed for CTLA-4^+^ cells count in the red pulp by immunohistochemistry.

### Qualitative analysis of the B cells expressing the exhaustion marker TIM-3 (CD21^+^TIM-3^+^) in the spleen of dogs naturally infected with *L*. *infantum*

**Figure 6:**
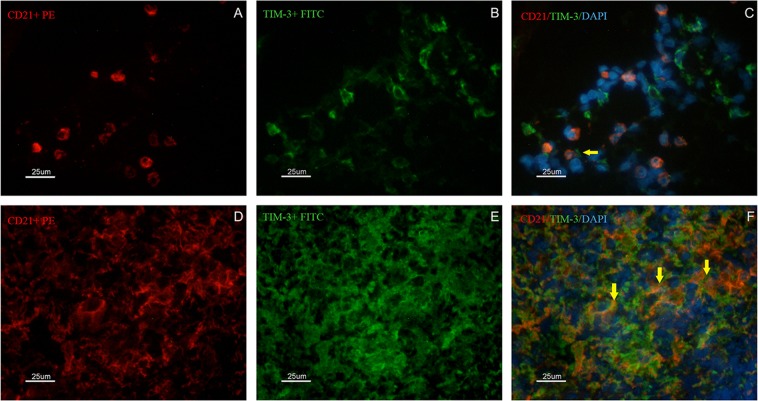
Identification of CD21^+^ and TIM-3^+^ cells by immunofluorescence in the spleen of dogs naturally infected by *L*. *infantum* presenting organized (**A**–**C**) or disorganized (**D**–**F**) splenic white pulp. In (**A**,**D**) the presence of CD21^+^ cells/B lymphocytes (red PE) is shown. In (**B**,**E**) the presence of TIM-3^+^ cells (green FITC) is shown. In (**C**,**F**) overlapping images (red PE/green FITC/blue DAPI) are shown. Yellow arrows indicate CD21^+^ cells also expressing TIM-3^+^. Scale bar: 25 μm.

The number of TIM-3^+^ cells was similar between the dogs with disorganized SWP and those with organized SWP, despite periarteriolar lymphoid sheath atrophy and a reduction in the number of CD4^+^ cells. Histopathological analysis revealed similar distributions of B lymphocytes in the OL, DL and DH groups. We questioned whether B cells could express TIM-3 in these dogs. Thus, double labelling was performed *in situ* using fluorescent antibodies. CD21^+^ B lymphocytes expressing the molecule TIM-3 were found (Fig. 6), and these cells were observed more frequently in the spleens with disorganized SWP.

### Quantitative and qualitative analysis of apoptotic cells in the spleen of dogs naturally infected with *L*. *infantum*

**Figure 7:**
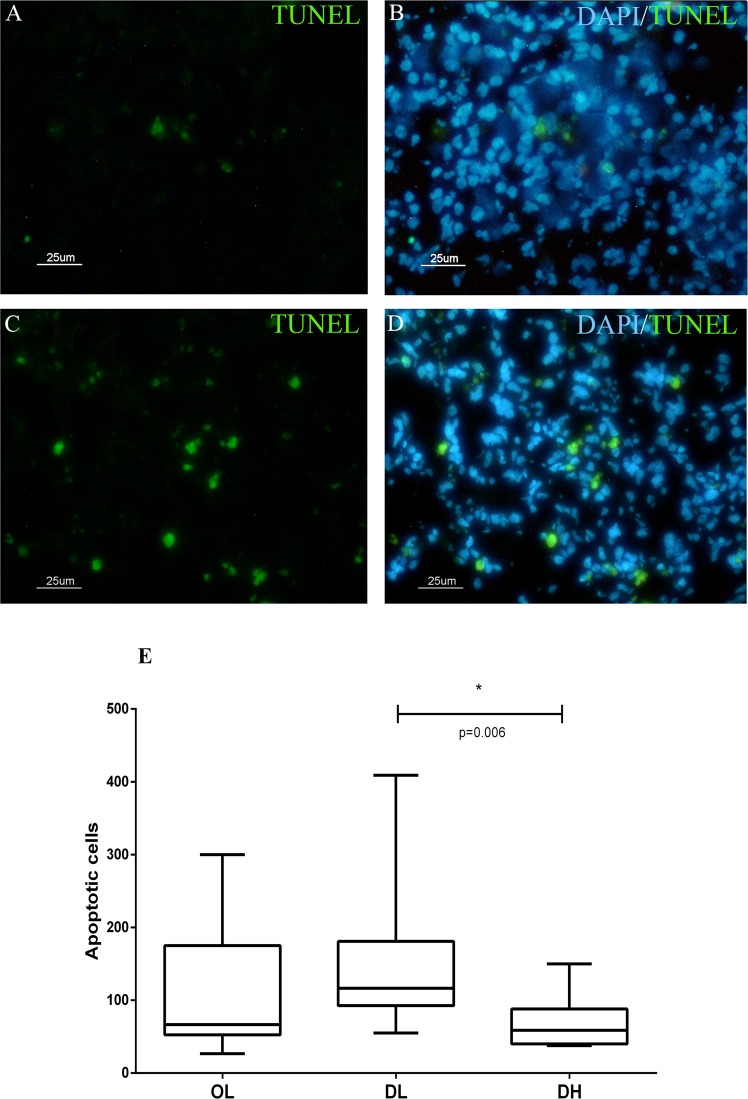
Identification of apoptotic cells by TUNEL staining of the spleen of dogs naturally infected by *L*. *infantum* presenting organized (**A**,**B**) or disorganized (**C**,**D**) splenic white pulp. Scale bar: 25 μm. (**E**) Quantitative analysis of apoptotic cells according to the disorganization of the splenic white pulp and the parasite load.

We observed fewer CTLA-4^+^ cells in the DH group than in the OL and DL groups. Acknowledging that the endpoint of cellular exhaustion is apoptosis, we hypothesized that these cells could be dying, leading to atrophy of the periarteriolar lymphatic sheath. Therefore, apoptotic cells in the spleens were analysed (Fig. 7A–D), identified by TUNEL staining, and quantified in both red pulp and white pulp. We found that apoptotic cells were homogeneously distributed throughout the SWP and red pulp.

**Figure 8:**
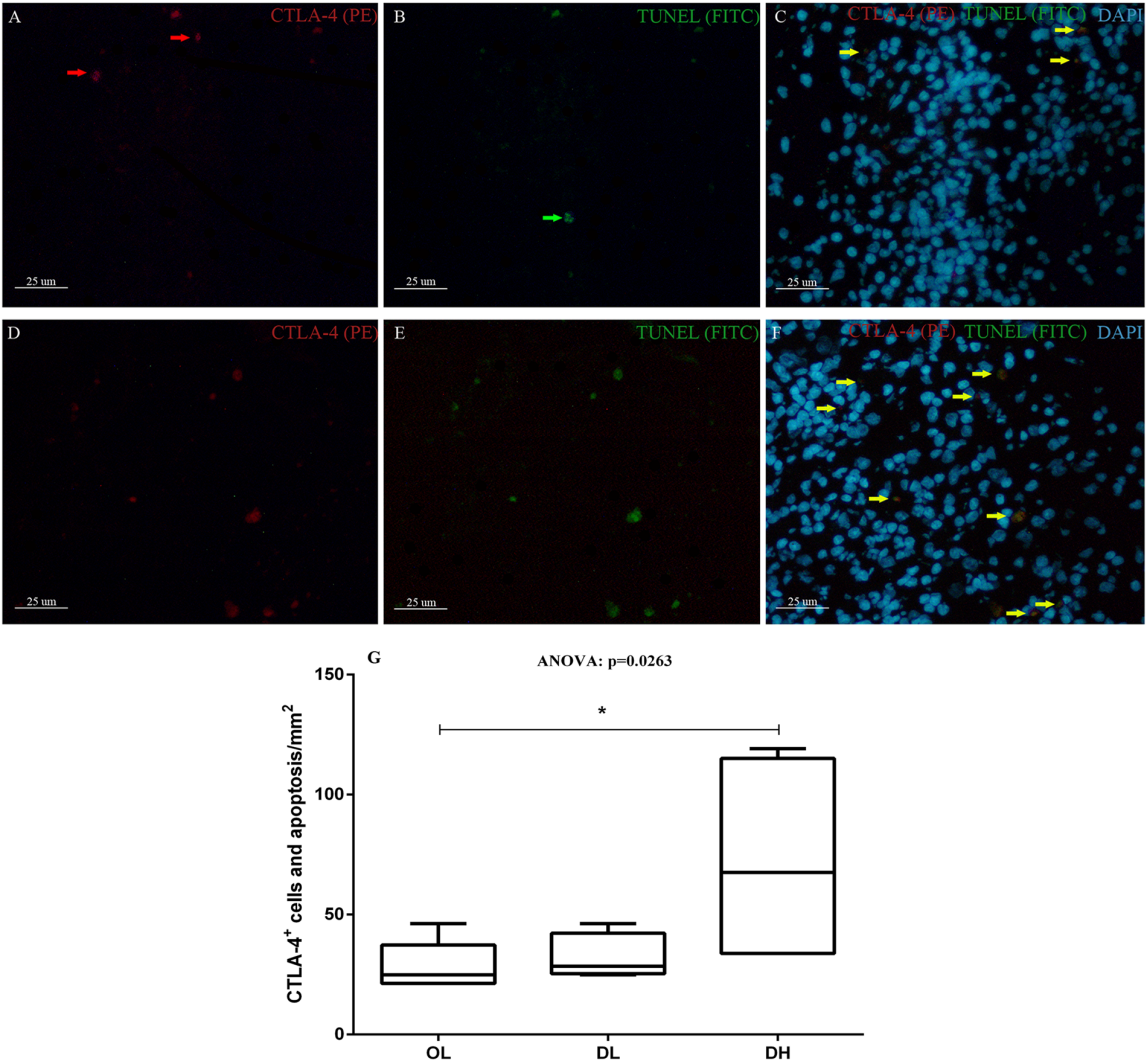
Identification of apoptotic CTLA-4^+^ cells by TUNEL staining of the spleen of dogs naturally infected by *L*. *infantum* presenting organized (**A**–**C**) or disorganized (**D**–**F**) splenic white pulp. Scale bar: 25 μm. Red arrows indicate isolated CTLA-4 expression. Green arrows indicate cells undergoing apoptosis without expressing CTLA-4. Yellow arrows indicate double labelling (CTLA-4^+^  + apoptosis). (**G**) Quantitative analysis of CTLA-4^+^ cells and apoptotic cells/mm^2^ according to the disorganization of the splenic white pulp and the parasite load. ANOVA: p = 0.0263. Mann-Whitney test (*): (**B**) p = 0.0303.

Although apoptotic cells could be visualized in the OL, DL and DH groups, they were more frequently observed in the animals with a low parasite load (Fig. 7E). We also performed double labelling for CTLA-4 and apoptosis marker expression in dogs in all groups, and the highest number of CTLA-4^+^ cells undergoing apoptosis was detected in the DH group (Fig. 8).

## Discussion

In the present study, we evaluated the spleens of 40 dogs naturally infected with *L*. *infantum*. Markers for the exhaustion phenotype were quantified, and the association of marker expression with clinical evaluation, organization of the SWP and parasite load were tested. The animals were grouped into low, medium and high clinical score groups. When these groups were compared, the number of IL-10^+^ cells was significantly lower in the animals with higher clinical scores (medium and high) than in the animals with low clinical scores. A previous study showed a reduction in the expression of IL-10 in animals with a high parasite load^6^. The reduction in IL-10 expression observed in the present study was associated with cellular exhaustion processes defined by the expression of different markers, such as PD-1, PD-L1, CTLA-4 and TIM-3. Interestingly, IL-10 has been related to VL progression in some studies^9,18,19^, while conversely, others have found no association^20,21^. In part, these conflicting results can be due to the model studied as well as the use of naturally infected animals, which prevents the verification of the time and evolution of the infection.

A study based on the PD-1 receptor showed an association among the cellular exhaustion process, a reduction in proliferative capacity and a decline in cytokine levels in the peripheral blood of polysymptomatic animals^22^. In another study using a mouse model chronically infected with lymphocytic choriomeningitis virus, the immune exhaustion process manifested in the early stages of infection, followed by increased expression of the PD-1 surface molecule induced by the pathogen^13^. It has been demonstrated in mice infected with *Plasmodium chabaudi*, *Plasmodium vinckei* and *Plasmodium berghei* that as soon as 6 days after infection, CD4^+^ T cells start to express PD-1 and LAG-3, suggesting that the inhibition of immune responses via these receptors may occur immediately after the onset of T cell activation^23^. Although ZVL was already established in the dogs evaluated in the present study, results similar to those reported in the abovementioned works were observed: the dogs with organized spleens and low parasite loads expressed considerable numbers of PD-1, PD-L1, PD-L2, CTLA-4 and LAG-3. Activation of PD-1 induces apoptosis and inhibits cell proliferation and cytokine production^15^. Nevertheless, in the present study, PD-1^+^ and LAG-3^+^ cells was not detected in splenic tissue by immunohistochemistry, which could be explained by the low resolution of this technique. It is also possible that antigenic re-stimulation of these cells for several days may be needed to enable the detection of the PD-1 and LAG-3 receptors *in situ*. Previous studies that identified exhausted cells in dogs used flow cytometry and re-stimulated the cells for a few days before using them in the analysis^22,24,25^. However, it has been shown that the expression of PD-L1 in canine oral melanoma, osteosarcoma, haemangiosarcoma, mast cell tumour, mammary adenocarcinoma and prostate adenocarcinoma can be identified by immunohistochemistry^26^. Human studies have reported a wide range of tissues that normally express PD-1 linker transcripts, with high levels of expression observed in the placenta, heart, lungs and liver and low levels of expression observed in the spleen, lymph nodes and thymus^27–30^. In this study, CTLA-4^+^ cells and the PD-1, PD-L1, PD-L2 and LAG-3 genes were expressed at lower levels in the groups with greater disorganization and higher parasite load in the spleen, with significant differences observed for CTLA-4, PD-L1 and LAG-3. In a study of a BALB/C mouse model infected with *L*. *donovani*, the authors observed an increase in CTLA-4^+^ expression in the initial stages of infection, 24–48 hours after infection^17^. In the present study, we observed fewer CTLA-4^+^ cells and decreased gene expression of the other exhaustion markers in the animals with disorganized SWP and high parasite load, possibly as a consequence of cell apoptosis, as the apoptotic profile was detected across all groups. The number of apoptotic cells was higher in the group presenting a low parasite load and in the group exhibiting disorganized white pulp than in the other groups. The lowest number of apoptotic cells was observed in the animals with disorganized spleen microarchitecture and high parasite load, a result similar to that observed for CTLA-4^+^ cells and the mRNA expression of PD-1 and LAG-3. In addition, we observed that the majority of apoptotic cells in the spleen of dogs in the DH group were CTLA-4^+^. This finding corroborates the hypothesis that the lower number of exhausted cells observed in the DH group is due to cell death with the substantial reduction in cellularity leading to immunosuppression. In macrophages, the PD-1/SHP2 signalling axis has been reported to negatively regulate the activation of AKT and that the H_2_O_2_-mediated induction of PD-1 expression contributes to apoptosis^31^. Currently, the involvement of CTLA-4 in the induction of apoptosis is not well defined. In a murine model, the binding of a monoclonal antibody (mAb) to the CTLA-4 receptor on the surface of activated T cells has been shown to induce apoptosis in a Fas-independent manner^32^. CTLA-4 can trigger apoptosis in CTLA-4-expressing tumour cells after the cells interact with soluble CD80 or CD86 recombinant ligands, and the induction of apoptosis occurs through a caspase-8-dependent mechanism^33^. In dogs infected with *L*. *infantum*, apoptosis has been observed in the lymph nodes and skin and is more frequently observed in organs with higher parasite loads^34^. The liver and spleen of infected dogs with *L*. *infantum* show a larger number of cells undergoing apoptosis (lymphocytes), suggesting that this process may contribute to the survival of *Leishmania* in these organs since lymphocytes undergoing apoptosis are not able to fight against parasites^34^. These results corroborate those of another study that observed apoptosis in T cells from the spleen and peripheral blood, suggesting that *Leishmania* parasites can induce apoptosis in T cells in association with a decrease in cell-mediated immunity^35^. Altogether, the data presented in this study suggest that apoptosis may be the mechanism involved in periarteriolar lymphatic sheath atrophy, lymphoid follicle atrophy, and the reduction in CD4^+^ and CTLA-4^+^ cell numbers.

The reduction in the number of CD4^+^ cells in the red pulp of animals in the DL group and the increase in these cells in the red pulp of animals in the DH group have been discussed in our previous work^7^. The accumulation of CD4^+^ cells in the red pulp of animals in the DH group was associated with atrophy of the periarteriolar lymphatic sheath. This finding suggests that these cells present defects in homing to their specific compartments in the white pulp and thus accumulate in the red pulp, where they eventually undergo apoptosis. In other studies, similar numbers of CD4^+^ cells were found in healthy and infected dogs, showing no correlation with clinical status^36^, which it may be indicative of the large individual variability among the dogs^37^. This reduction in cellularity was also evidenced by the reduced numbers of cells isolated from the spleen in dogs in the DH group and by the detection of periarteriolar lymphatic sheath and lymphoid follicle atrophy. T lymphocytes were few in number or absent from the white pulp and could be found in red pulp, which represents most of the remaining tissue. However, the number of B lymphocytes and plasma cells increased and accumulated in this area^38^, which may contribute to the total number of apoptotic cells in the DL and DH groups. Finally, we also detected CD4^+^ and CD8^+^ cells expressing both TIM-3 and CTLA-4 exhaustion receptors. However, no significant differences were found among the groups. When analysing naturally infected dogs, a high degree of variability is inevitable, which can influence the results. However, the DL group presented the highest CD4^+^CTLA-4^+^ counts, similar to that observed for the CTLA-4^+^ cell count in the red pulp by immunohistochemistry.

Interestingly, despite lower quantities of T lymphocytes, as measured by periarteriolar lymphatic sheath atrophy and the detection of apoptotic cells, we observed that the expression of TIM-3 was high in all groups. We hypothesized that exhaustion receptors are expressed on cells other than T lymphocytes—possibly B lymphocytes, as B lymphocytes represent a frequently observed subpopulation in the spleen. To answer this question, we identified CD21^+^TIM3^+^ B cells in the spleen of infected dogs, suggesting that the cellular exhaustion process may also occur in B cells during the parasitism of dogs by *L*. *infantum*. Studies examining exhausted B lymphocytes expressing TIM-4^+^ have already been performed in mice^39^, but the role of TIM-3 in leishmaniasis has not yet been clarified. In dogs infected by *L*. *infantum*, the association of SWP microarchitecture disruption with the accumulation of plasma cells in the spleen has been demonstrated. In addition, dogs with active infection and disorganization of the white pulp present more severe dysproteinaemia and an increased serum globulin fraction, which also correlates with the intensity of the accumulation of plasma cells^40^. Thus, IgG-producing cells are predominantly responsible for the increase in plasma cell density in the spleen^40^. In addition, the expression levels of CXCL12, APRIL and BAFF, molecules associated with the localization and survival of plasma cells^41^, are elevated in the splenic tissue of animals with active infection and disorganization of the SWP^40^. Thus, it is reasonable to speculate that polyclonal activation of B cells contributed to diminished apoptotic cells in DH dogs. Plasmacytosis in secondary lymphoid organs has been proposed to be primarily responsible for the hypergammaglobulinaemia associated with chronic inflammatory diseases^42^. Moreover, splenic plasmacytosis has been reported to be strongly associated with high serum globulin concentrations, a relative increase in the serum gamma globulin fraction and serum dysproteinaemia^40^. Serum protein electrophoresis has revealed that the hypergammaglobulinemia presented by animals with infection and the disorganization of the SWP are clearly distributed polyclonally^43^. The role of PD-L1 in B cell function and experimentally induced chronic inflammatory responses in dogs has been demonstrated, and the presence of a novel and critical molecule for B cell regulation, the B10 regulator, has been shown to induce the expression of IgD during progressive VL^44^. Cells expressing B10 produce IL-10, which induces other B cells and T cells to produce IL-10 and suppresses IFN-γ through PD-L1/PD1. PD-L1^+^ cell types were not characterized in the present study, although PD-L1 gene expression was detected. Despite these published data, the role of exhausted B cells in the immunopathogenesis of VL requires further clarification.

Splenic disorganization caused by *L*. *infantum* infection and/or coinfections appears as rupture of the splenic microarchitecture, hindering the activation of lymphocytes and the immune response against antigens and thereby suppressing the immune response against *Leishmania* and other pathogens. Our data support the hypothesis that the alteration of the extracellular matrix of the spleen is related to the cellular exhaustion process and, consequently, to immunosuppression. This hypothesis may explain the development of clinical signs and the failure to control the parasite load in animals with SWP disorganization. We observed that most of the dogs presented disorganization of the microarchitecture of the SWP (N = 30, 73.2%). Additionally, we observed reductions in the size and number of lymphoid follicles, the number of cells in the periarteriolar lymphatic sheath, and the quantity of CD4^+^ lymphocytes in the red pulp. These results indicate that T and B lymphocytes may not migrate to their specific sites and/or are dying by apoptosis. According to the present data and the data reported by other authors^35^, the association between a high percentage of T cell apoptosis and structural disorganization of the SWP contributes to the inefficient cell-mediated immune response in dogs infected by *L*. *infantum*. The presence of apoptotic cells has also been reported, and naturally infected dogs have been demonstrated to exhibit a decreased total population of T lymphocytes and an increased percentage of apoptotic cells in the spleen and peripheral blood^4^.

Our results indicate that T and B cell exhaustion occurs in the spleen of dogs naturally infected with *L*. *infantum*. To the best of our knowledge, this is the first description of the expression of CTLA-4, TIM-3 and LAG-3 in the spleen of naturally infected dogs. The high expression levels of these markers indicate that they should be considered in approaches involving exhaustion process blockade, with immunomodulation of molecular pathways as an alternative therapeutic strategy for dogs with ZVL. Ultimately, we demonstrated associations among cellular exhaustion, apoptosis, splenic disorganization, reduction in CD4^+^ lymphocyte numbers, failure to control the parasite load and the worsening of disease.

## Methods

### Ethics statement

The animals included in this study were dogs naturally infected with *L*. *infantum* that were destined for euthanasia, as recommended by the policies of the Brazilian Ministry of Health. All dog owners provided formal written consent for study participation. The samples were collected during necropsies conducted by veterinarians from the Laboratório de Pesquisa Clínica em Dermatozoonoses em Animais Domésticos (LAPCLIN-DERMZOO-INI/FIOCRUZ). This study was approved by Comitê de Ética em Uso de Animais (CEUA-Fiocruz) under the licence LW-54/13 and conducted according to the Brazilian Law 11794/08 and Sociedade Brasileira de Ciência em Animais de Laboratório (SBCAL).

### Animals

Forty dogs from Barra Mansa, Rio de Janeiro, Brazil, with a confirmed diagnosis of infection by *L*. *infantum* referred for compulsory euthanasia at the Evandro Chagas National Institute of Infectious Diseases (INI/FIOCRUZ) were evaluated. Euthanasia was performed by the veterinarians as follows: a) 1.0% (1.0 mL/kg) thiopental (Thiopentax®, Cristalia) was administered intravenously; and b) after the detection of the absence of a corneal reflex, which was induced by deep anaesthesia, 10 mL of 19.1% potassium chloride (Isofarma) was administered intravenously. The biological samples were obtained (the spleen and peripheral blood) during necropsy, and clinical data were scored prior to necropsy by two veterinarians.

### Clinical evaluation of the animals

Clinical evaluation was performed by two veterinarians who assessed the 6 typical clinical signs of ZVL in dogs: dermatitis, onychogryphosis, conjunctivitis, loss of body condition, alopecia and lymphadenomegaly. Each clinical sign was evaluated on the following scale: 0 (absent), 1 (mild), 2 (moderate) and 3 (severe)^20^. The final classification was calculated as the sum of the points obtained; the animal could have a low clinical score (0 to 2 points), medium clinical score (3 to 6) or high clinical score (7 to 18).

### Histopathological analysis

Tissue fragments from the spleen were fixed in 10% formalin-buffered solution (Merck, Darmstadt, Germany) and embedded in paraffin (Synth, Diadema, Brazil). Histological sections (5 μm thick) were affixed to microscopic slides and stained with haematoxylin and eosin (Leica Biosystems, Newcastle Upon Tyne, UK) for further analysis under an optical microscope (Zeiss, Oberkochen, Germany). Organization of the splenic lymphoid tissue of the white pulp, marginal zone and red pulp was analysed as previously described^3^. The white pulp was scored as follows: 1- organized with periarteriolar sheath, germinal centres, and distinct mantle and marginal zone. 2- Slightly disorganized, with some hyperplastic or hypoplastic change leading to the loss of definition of some regions of the white pulp. 3- Moderately disorganized, with evident white pulp, but the regions are barely individualized or indistinct. 4- Intensely disorganized, with a follicular structure little distinguishable from the red pulp and the T cell area. Quantification of the number of lymphoid follicles per mm^2^ of tissue was also performed. The evaluation considered the presence or absence of follicles as well as the number of lymphoid follicles per field.

### Extraction of DNA from splenic tissue

Total DNA was isolated from approximately 10 mg of spleen using the QIAmp DNA Mini Kit (Qiagen, Santa Clarita, USA), which included an early digestion with proteinase K (20 mg/mL) for 1 hour at 56 °C. The DNA was eluted in TE buffer and quantified with a NanoDrop® spectrophotometer (Thermo Fisher Scientific, Waltham, USA).

### Determination of the parasite load by qPCR

The parasite load in the spleen was estimated by real-time PCR as previously described^6^. HPRT primers (Supplementary Table 1) were used to normalize the canine DNA concentrations in each sample. To quantify the number of DNA copies pertaining to the parasites, the primers for ssrRNA (Supplementary Table S1) were used^45^ to amplify the gene encoding the ribosomal minor subunit RNA (ssrRNA, multi-copy gene). qPCR assays were performed with a Step One instrument (Applied Biosystems, Molecular Probes, Inc., Foster City, USA) using Power SYBR Green Master Mix (Applied Biosystems, Molecular Probes, Inc., Foster City, USA). Two microliters of the purified total DNA (100 ng) was added to a final PCR volume of 20 μl containing Power SYBR Green 1X and 300 nM of each primer for HPRT PCR assays or 500 nM of each primer for ssrRNA PCR assays. qPCR was performed with an activation step at 95 °C for 10 minutes, followed by 40 cycles of denaturation and annealing/extension (95 °C for 15 seconds, 60 °C for 1 minute and 68 °C for 30 seconds). A standard curve was created for each target. All reactions were performed in duplicate for each target, and both targets were run on the same plate for the same sample. Peripheral blood mononuclear cells (PBMCs) from uninfected dogs and bulk cultures of *L*. *infantum* promastigotes (MCAN/BR/2007/CG-1) were quantified using a cell counter (Z1™ COULTER COUNTER®, Beckman Coulter, Fullerton, USA). Total DNA was extracted from 1.0 × 10^6^ PBMCs and 1.0 × 10^7^ promastigotes. Standard curves for the HPRT and ssrRNA genes were prepared using 10-fold serial dilutions. The animals were grouped into high or low parasite load groups as previously described^6^.

### RNA extraction and quantification of the gene expression of exhaustion markers (PD-1, PD-L1, PD-L2 and LAG-3) by qRT-PCR

Total RNA was extracted from spleen tissue fragments using Trizol reagent (Invitrogen, Grand Island, NY), followed by final purification with an RNeasy kit (Qiagen) according to the manufacturer’s protocol. RNA was quantified with a Nanodrop TM 1000 spectrophotometer (Thermo Fisher Scientific Inc., Waltham, USA), and 2 μg of RNA was treated with RNase-free DNase (Ambion, Grand Island, NY). cDNA synthesis was performed with a High Capacity cDNA Synthesis kit (Applied Biosystems), and a control for genomic DNA contamination was included. qPCR was performed using a Power SYBR Green Master Mix® system (Applied Biosystems, Molecular Probes, Inc.) on a ViiA 7 instrument (Applied Biosystems, Molecular Probes, Inc.). Reactions occurred in a final volume of 10 μl. Primer sequences and concentrations are shown in Supplementary Table S2. The thermal cycle consisted of an activation step at 95 °C for 10 minutes, followed by 45 cycles of denaturation at 95 °C for 15 seconds and annealing/extension at 60 °C for 1 minute. A dissociation curve (from 60 °C to 95 °C) was created for non-specific amplification evaluation. For the PD-1 and LAG-3 sets, a reading step was added at 80 °C and 78 °C, respectively, shortly after the annealing/extension step. Normalized expression of the genes of interest was previously determined^46^, where HPRT and GAPDH^6^ were used as references. Efficiencies were determined experimentally with serial dilutions of dog cDNAs^47^. All reactions were performed in duplicate, and negative controls were included in all trials.

### Immunohistochemistry

Tissue fragments from the spleen were frozen in TissueTek OCT resin (Sakura, Alphen aan den Rijn, The Netherlands). Spleen sections with a thickness of 5 μm were fixed with acetone P.A. (Merck) on silanized slides (Dako, Carpinteria, CA, USA). Then, the sections were hydrated in phosphate-buffered saline (PBS) for 10 minutes, followed by inhibition of endogenous peroxidase with a solution containing 3% hydrogen peroxide (Dako) for 1 minute at room temperature. The sections were washed twice in PBS for 5 minutes each time. For the inhibition of non-specific binding, the sections were incubated in a solution containing 0.4% BSA for 20 minutes at room temperature. The excess blocking solution was discarded, and primary antibodies for CD4^+^ (YKIX302.9), CD8^+^ (YCATE55.9), CD21^+^ (CA2.1D6) (Bio-Rad AbD Serotec, Hercules, USA), the cytokines IFN-γ (CC302) and IL-10 (CC318) (Bio-Rad AbD Serotec), the proliferation marker Ki-67 (SolA15) (eBioscience, San Diego, USA), and the exhaustion markers PD-1 (NAT105), LAG-3 (11E3), CTLA-4 (BNI3) and TIM-3 (polyclonal) (Abcam, Cambridge, UK) were added for 18 hours at 4 °C. Control of the reaction was accomplished by suppressing the primary antibody in at least one of the tissue sections. The slides were subsequently washed with PBS twice for 5 minutes each and then incubated with the appropriate biotinylated secondary antibody (Dako) for 25 minutes, followed by another 2 washes with PBS. The sections were incubated with streptavidin-peroxidase (Genetex) for 25 minutes. After another 2 washes with PBS, the antigen-antibody reaction was detected with an AEC kit (Invitrogen, Carlsbad, USA). After visualization of the optical microscopy marking, the reaction was stopped with type II water, and the sections were counterstained with Meyer’s haematoxylin (Sigma-Aldrich, Saint Louis, USA), followed by assembly in Faramount medium (Dako). Five slides per animal were evaluated under an optical microscope, and the levels of the lymphocyte markers CD4^+^, CD8^+^, and CD21^+^; cytokines; the proliferation marker Ki-67; and exhaustion markers were quantified in the splenic red pulp in alternating fields under 40x magnification. The results were expressed as the percentage of positive cells and cells/mm^2^. Qualitative analyses were performed in both the red and white pulp.

### Immunofluorescence

The splenic tissue was cryosectioned at a thickness of 5 μm, and the sections were placed on silanized slides (Dako, Carpinteria, CA, USA) and fixed with acetone P.A. (Merck) for 10 minutes. For the inhibition of non-specific binding, the sections were incubated in a solution containing 0.4% BSA for 20 minutes at room temperature. The excess blocking solution was discarded, and the primary antibodies for the detection of CD21 (Bio-Rad AbD Serotec), CTLA-4 and TIM-3 (Abcam) were added for 18 hours at 4 °C. The reaction was revealed by a secondary anti-mouse IgG antibody conjugated to phycoerythrin (PE-red) and an anti-rabbit IgG antibody conjugated to fluorescein isothiocyanate (FITC). The incubation with the secondary antibodies was performed in a dark room for 30 minutes at room temperature. The slides were assembled using Fluoromount-g medium containing DAPI (4′, 6-diamino-2-phenylindole) (Thermo Fisher Scientific), and the reaction was observed under a fluorescence microscope. The images were processed and overlaid with ImageJ software (NIH, USA).

### Tunel

Silanized slides containing spleen sections were fixed in 10% paraformaldehyde in PBS for 15 minutes at room temperature. They were then washed twice in PBS for 5 minutes each. Cell permeabilization was performed with proteinase K (Invitrogen, Carlsbad, CA, USA, final concentration of 20 μg/mL) in 10 mM Tris/HCl pH 8.0 reconstituted (10 mg/mL) and diluted 1:500 in PBS. A volume of 100 μl of proteinase K (20 μg/mL) was added to cover the section of tissue for 10 minutes at room temperature. The slides were washed in PBS, and the TUNEL reagent (DeadEnd™ Fluorometric TUNEL assay, Promega Co., USA) was added to the sections according to the manufacturer’s instructions. This reagent allows the detection of DNA fragmentation by labelling the terminal portions of nucleic acids. For the negative control, only the equilibration buffer was added. Incubation in a humid chamber was performed at 40 °C for 90 minutes. The slides were subsequently washed with PBS for 5 minutes under constant stirring, and after drying, they were mounted in Fluoromount-G with DAPI (Thermo Fisher Scientific, USA). The slides were analysed under a Zeiss fluorescence microscope, and the labelled apoptotic cells were quantified to their full extent in alternating fields under 40x magnification. The total number of DAPI-labelled cells was also quantified.

### Flow cytometry

Splenocytes were isolated from splenic fragments 1 cm in diameter by mechanical disruption, followed by Histopaque gradient (Sigma). Splenocytes were subsequently frozen in 90% FCS and 10% DMSO (Sigma) until use. Frozen splenocytes were thawed at 37 °C and washed in PBS/10% foetal bovine serum and quantified using a Neubauer chamber. Only samples with 60% cell recovery and 85% viability were evaluated. Each well was plated with 1 × 10^6^ cells and then incubated for 15 minutes in blocking solution containing 5 mL of human serum per well. Thereafter, the anti-CD4 (YKIX302.9), anti-CD8 (YCATE55.9) (Bio-Rad AbD Serotec, USA), anti-CTLA-4 (BNI3) and anti-TIM-3 (polyclonal) (Abcam, United Kingdom) primary antibodies were incubated for 30 minutes and subsequently washed with PBS and plate centrifuged. The secondary antibodies anti-rabbit FITC (eBioscience, San Diego, USA), anti-mouse Dylight 633 (ImmunoReagents, Raleigh, USA) and anti-mouse Dylight 488 (KPL, Gaithersburg, USA) and isotypes APC and FITC (eBioscence, USA) were added, followed by lavage and fixation. Data were acquired with a BD Canto II flow cytometer using BD software FACSDiva (BD Bioscience, USA). Automatic compensation was performed at the beginning of each experiment, and data were analysed using the FlowJo v10 program (TreeStar Software, USA).

### Statistical analysis

This study used a non-probabilistic sample, and for analysis purposes, the animals were divided into 3 groups according to their clinical signs: 1- low clinical score; 2- medium clinical score; and 3- high clinical score. For the analysis, the animals were also divided according to the organization of the splenic lymphoid tissue and the parasite load: 1- organized/low load; 2- disorganized/low load; and 3- disorganized/high load. The non-parametric Mann-Whitney and Kruskal-Wallis tests for independent samples and the Spearman rank correlation test were performed using GraphPad Prism version 5.0 (GraphPad Software, San Diego, CA, USA). For gene expression analyses, ANOVA and Tukey tests were used. Values of P < 0.05 were considered significant. Data are represented by the median and interquartile range.

## Supplementary information

Supplementary information
