# Supplementary material for: Pro-Cellular Exhaustion Markers are Associated with Splenic Microarchitecture Disorganization and Parasite Load in Dogs with Visceral Leishmaniasis
Source: Sci Rep. 2019 Sep 10;9:12962. doi: 10.1038/s41598-019-49344-1 (PMC6736856; doi:10.1038/s41598-019-49344-1)

Pro-cellular exhaustion markers are associated with splenic microarchitecture disorganization and parasite load in dogs with visceral leishmaniasis

Tainã Luís de Souza[1], Aurea Virginia Andrade da Silva[1], Luiza de Oliveira Ramos Pereira[1], Fabiano Borges Figueiredo[2], Artur Augusto Velho Mendes Junior[3], Rodrigo Caldas Menezes[3], Daniella Areas Mendes-da-Cruz[4], Mariana Côrtes Boité[1], Elisa Cupolillo[1], Renato Porrozzi[1], Fernanda Nazaré Morgado[1]

[1] Laboratório de Pesquisa em Leishmaniose, Instituto Oswaldo Cruz, Fundação Oswaldo Cruz, Rio de Janeiro, Brazil.

[2] Laboratório de Biologia Celular, Instituto Carlos Chagas, Fundação Oswaldo Cruz, Paraná, Brazil.

[3] Laboratório de Pesquisa Clínica em Dermatozoonoses em Animais Domésticos, Instituto Nacional de Infectologia Evandro Chagas, Fundação Oswaldo Cruz, Rio de Janeiro, Brazil.

[4] Laboratório de Pesquisas Sobre o Timo, Instituto Oswaldo Cruz, Fundação Oswaldo Cruz, Rio de Janeiro, Brazil.

Supplementary Table 1

Target genes and their sequences used in quantification of the parasite load. S, sense; AS, antisense, bp, base pairs

Target Gene	Access number or reference		Sequence (5'-3')	Base pair size (bp)
HPRT<br>Canine target	NW_003726126.1	S	AAAACAATGCAGACTTTGCT	58
		AS	CCTTGACCATCTTTGGATTA	
SsrRNA<br>Parasite target	Prina et al. (2007)	S	TACTGGGGCGTCAGAG	153
Leishmania (L). infantum		AS	GGGTGTCATCGTTTGC	

Supplementary Table 2

Target genes and primer design for cDNA. S, sense; AS, antisense, bp, base pairs

Target gene	Access number		Sequence (5'-3')	Base pair size (bp)
HPRT	NW_003726126.1	S	AAAACAATGCAGACTTTGCT	58
		AS	CCTTGACCATCTTTGGATTA	
GAPDH	XM_003434387.2	S	ATGCCCAACATTGGTTATGG	103
		AS	CTCTTTCCACGATGGCTTTG	
PD-1	XM_022409577.1	S	TCCACGAACACTCAGAAGCC	136
		AS	TTCCTACTCCCCTTCAGGCA	
PD-L1	AB_850883.1	S	CCGCCAGCAGGTCACTTC	139
		AS	CCATTGTCACATTGCCACCA	
PD-L2	XM_014117874.2	S	CCTTGAACTGGTGTGGCAGA	132
		AS	TGCACTCCCAGGCTCAAAAT	
LAG-3	XM_005637438.2	S	AGTCATCACAGTGACTCCCA	132
		AS	GAACCTCGCCAAGACTGCTT	

Supplementary Figure S1

Representative images of the splenic white pulp (SWP) of dogs naturally infected with *L. infantum*. (A) Organized SWP, **(B)** slightly disorganized SWP, **(C)**, SWP with medium disorganization, and **(D)**, intensely disorganized SWP. Scale bar 10 µm. SWP = Splenic White Pulp; GC = Germinal Centre; CA = Central Artery; PLS = Periarteriolar Lymphatic Sheath; and SRP = Splenic Red Pulp. **(E)** Clinical score observed according to the degree of organization of the SWP. Mann-Whitney test: *p=0,0235. **(F)** Quantitative analysis of the dogs in the different groups according to the clinical score and splenic disorganization/parasite load. Organized SWP: Animals with SWP organized to slightly disorganized. SWP disorganized: animals with medium to intense disorganization of the SWP.

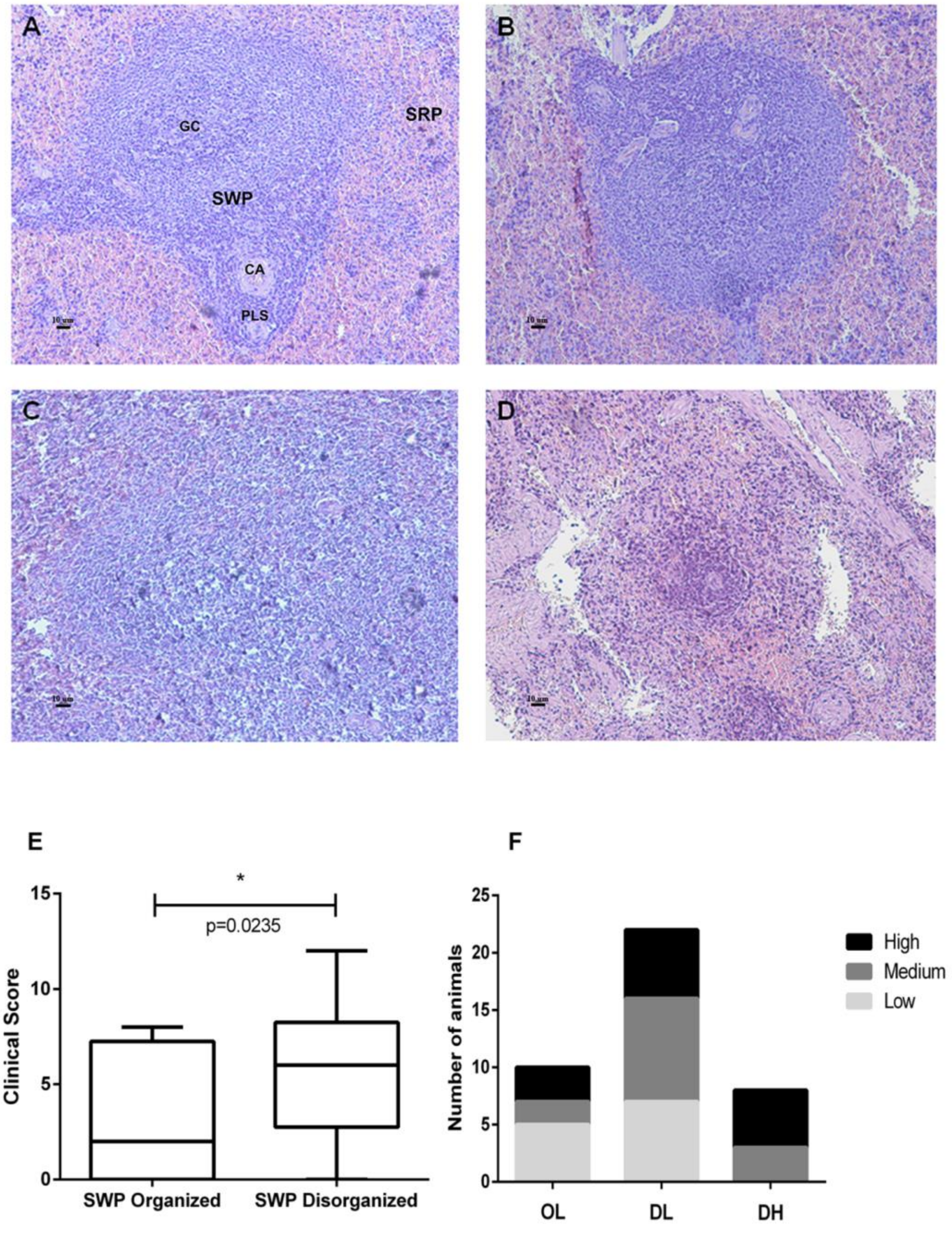

Supplementary Figure S2

Cell profile observed in the spleen of dogs naturally infected with *L. infantum* according to the clinical score: (A) percentage of $CD4^+$ cells, **(B)** $CD4^+$ cells/mm^2, **(C)** percentage of $CD8^+$ cells, **(D)** $CD8^+$ cells/mm^2, **(E)** percentage of $CD21^+$ cells and **(F)** $CD21^+$ cells/mm^2. Mann-Whitney and Kruskal-Wallis tests: $p>0.05$.

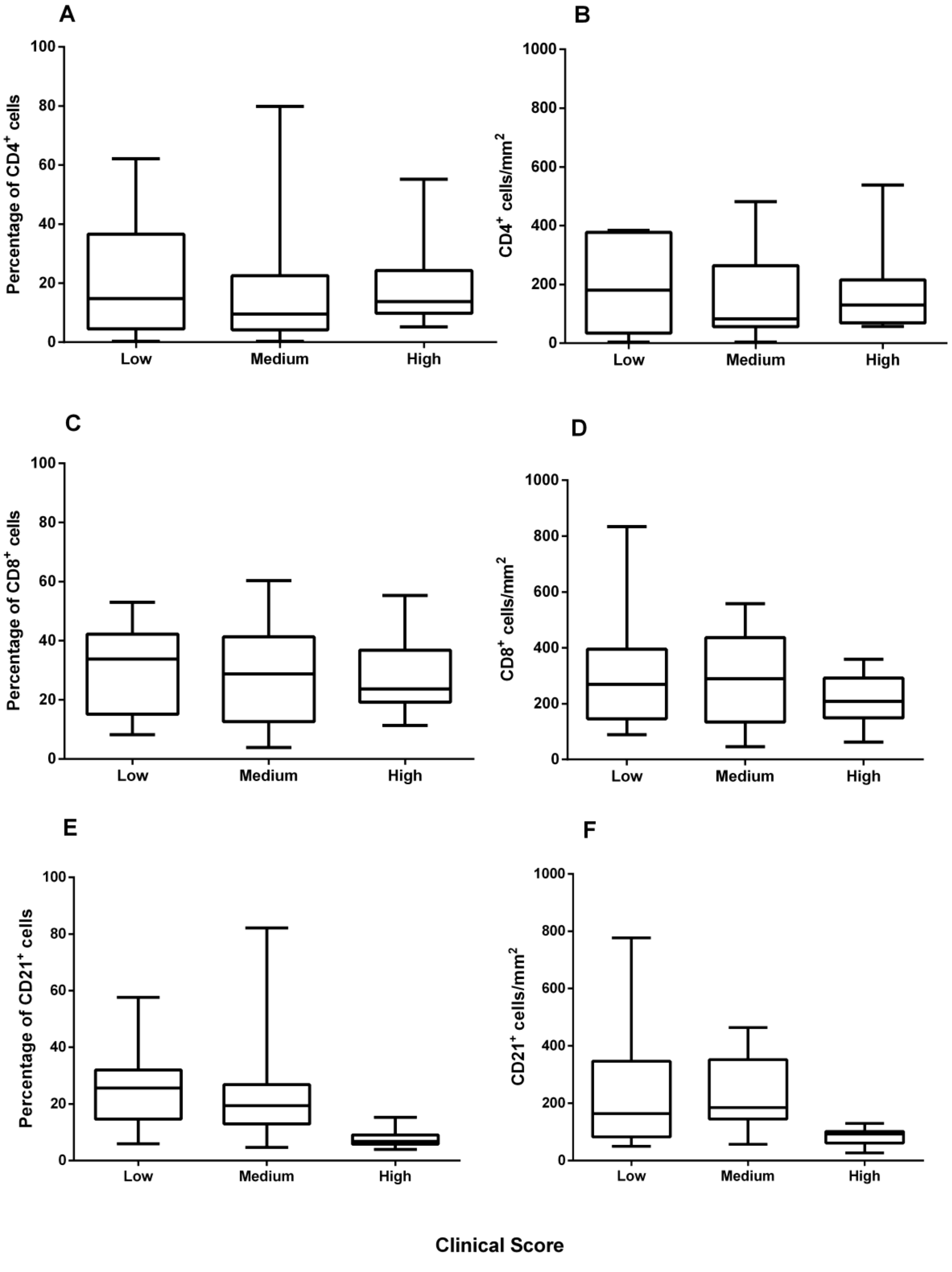

Supplementary Figure S3

Cell profile observed in the spleen of dogs naturally infected by *L. infantum* grouped according to the disorganization of the white splenic pulp and the parasite load: (A) percentage of IFN-γ$^+$ cells, **(B)** IFN-γ$^+$/mm^2 cells, **(C)** percentage of IL-10$^+$ cells, **(D)** IL-10$^+$ cells/mm^2, **(E)** percentage of Ki-67$^+$ cells, and **(F)** Ki-67$^+$ cells/mm^2. Mann-Whitney and Kruskal-Wallis tests: $p>0.05$.

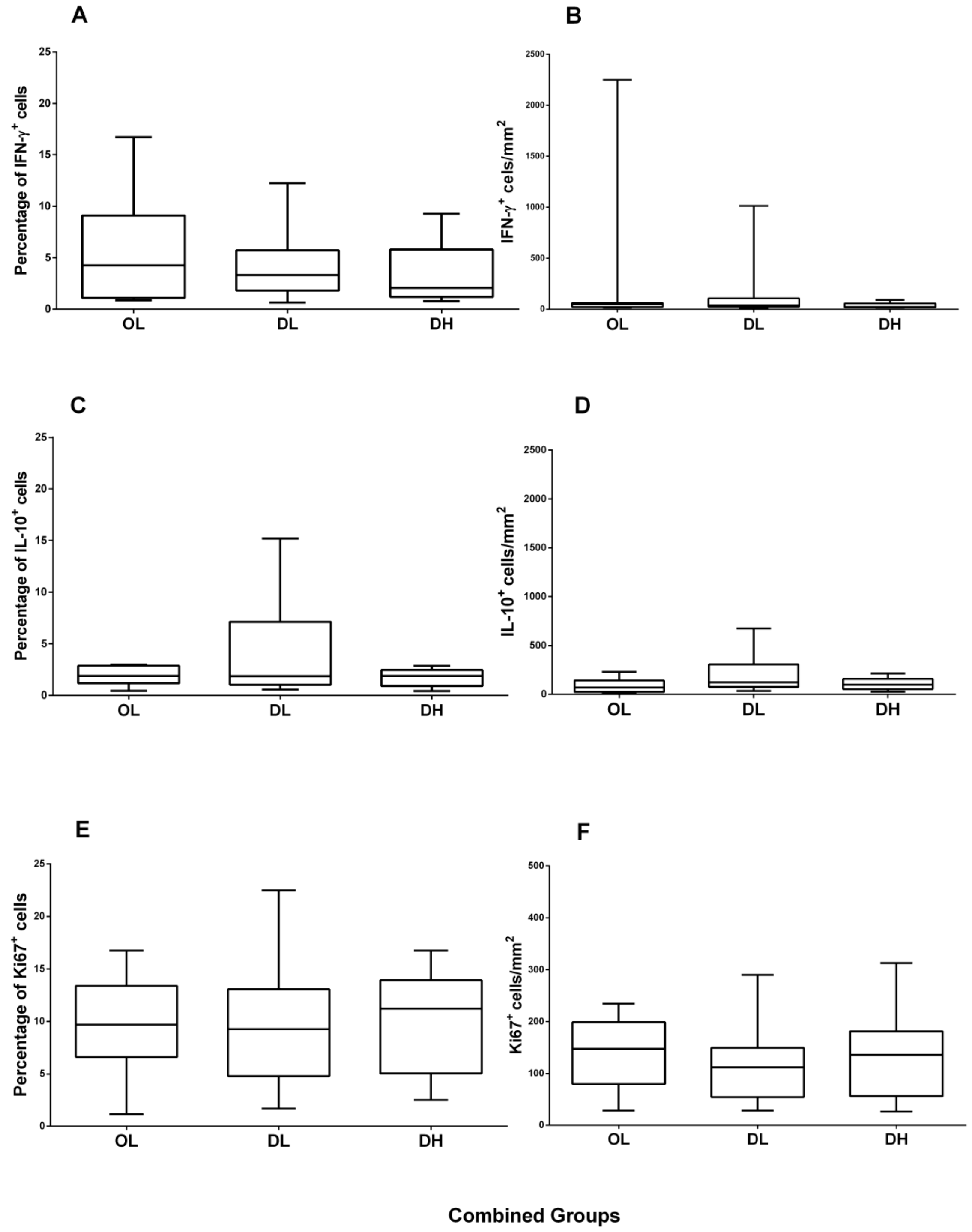

Supplementary Figure S4

Cellular exhaustion profile observed in the spleen of dogs naturally infected with *L. infantum* according to the clinical score: (A) percentage of TIM-3$^+$ cells, **(B)** TIM-3$^+$/mm^2, **(C)** percentage of CTLA-4$^+$ cells, and **(D)** CTLA-4$^+$ cells/mm^2. Mann-Whitney and Kruskal-Wallis tests: $p>0.05$.

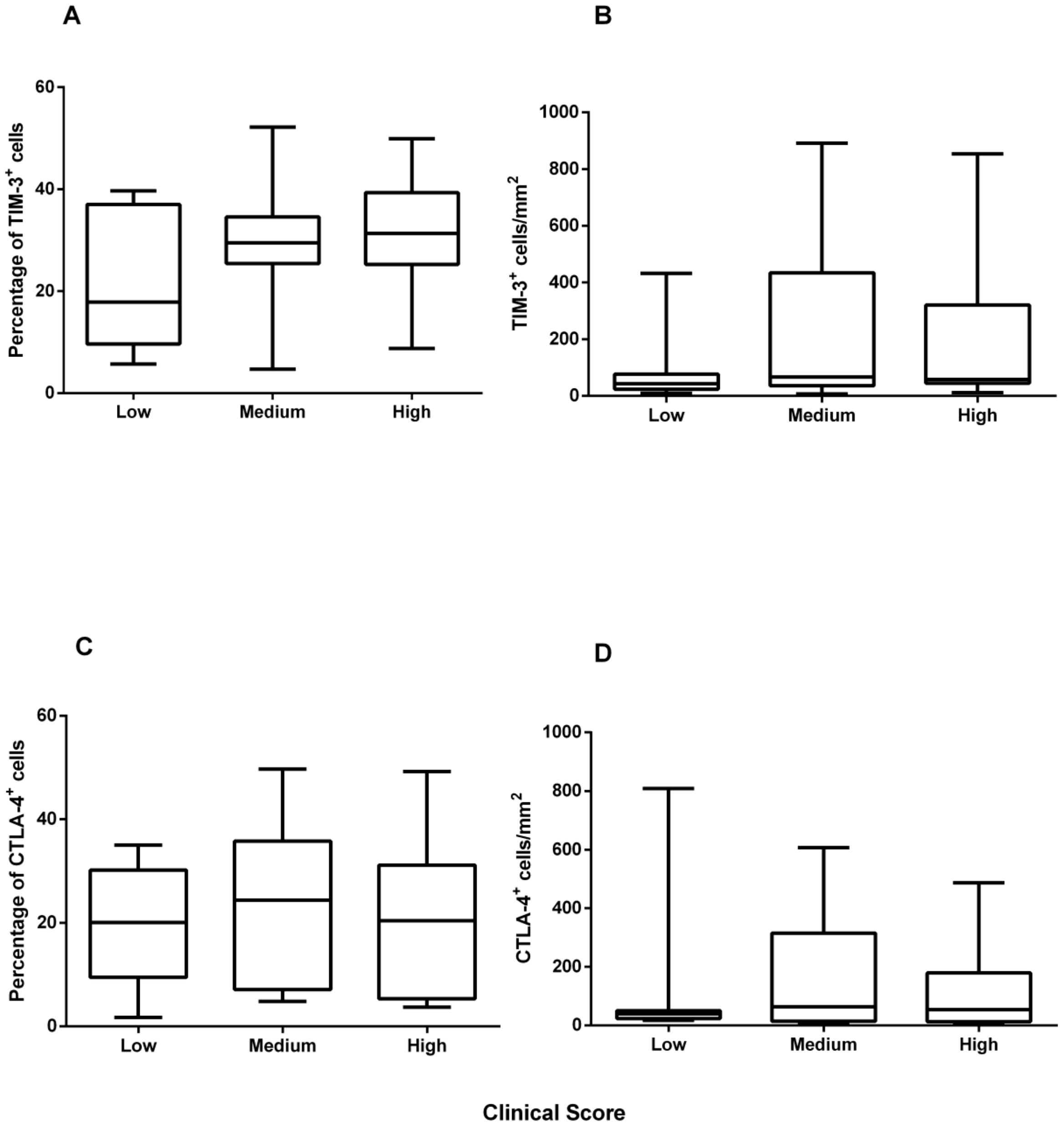

Supplementary Figure S5

Quantitative analysis by flow cytometry of CD4$^+$ and CD8$^+$ subpopulations expressing the exhaustion receptors TIM-3 and CTLA-4. (A) Representative images of lymphocyte gates, histograms of CTLA-4- and TIM-3-positive cells. **(B)** Percentage of CD4$^+$ cells, **(C)** CD4$^+$CTLA-4$^+$ cells, **(D)** CD4$^+$TIM-3$^+$ cells, **(E)** CD8$^+$ cells, **(F)** CD8$^+$CTLA-4$^+$ cells, **(G)** CD8$^+$TIM-3$^+$ cells among groups. OL: organized SWP and low parasite load; DL: disorganized SWP and low parasite load; DH: disorganized SWP and high parasite load.

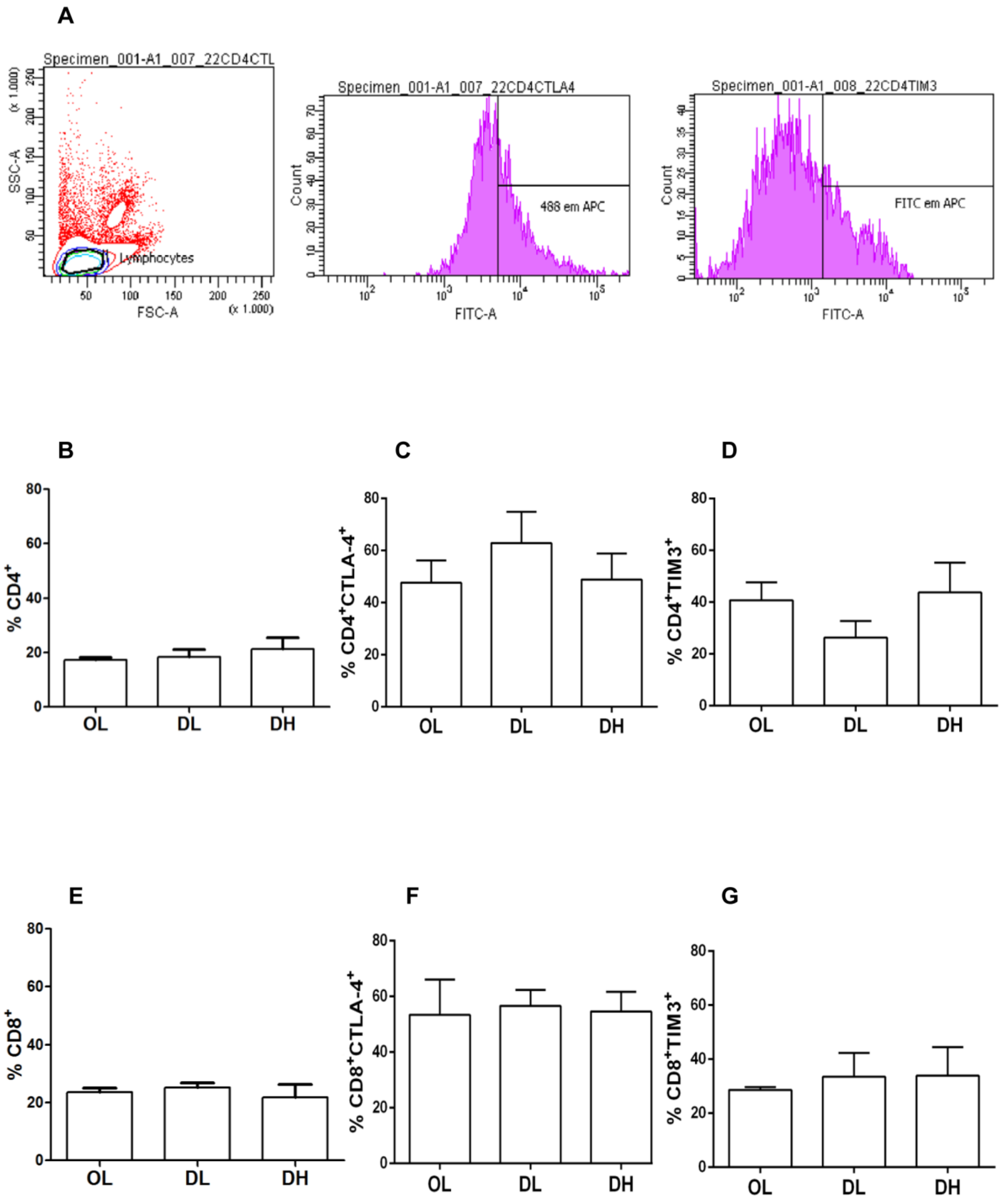

Supplement: Supplementary file 1 — Supplementary information [file 41598_2019_49344_MOESM1_ESM.pdf]
